# Endplate and intervertebral disc injuries in acute and single level osteoporotic vertebral fractures: is there any association with the process of bone healing?

**DOI:** 10.1186/s12891-019-2719-5

**Published:** 2019-07-19

**Authors:** Tatsuhiko Fujiwara, Koji Akeda, Junichi Yamada, Tetsushi Kondo, Akihiro Sudo

**Affiliations:** 1 Department of Orthopaedic Surgery, Murase Hospital, 3-12-10 Kanbe, Suzuka City, Mie 514-0801 Japan; 2 Department of Orthopaedic Surgery, Mie University Graduate School of Medicine, 2-174 Edobashi, Tsu City, Mie 514-8507 Japan

**Keywords:** Endplate injury, Intervertebral disc injury, Osteoporosis, Vertebral fracture

## Abstract

**Background:**

The endplate-intervertebral disc (IVD) complex is closely interrelated with the vertebral body (VB) in the structural integrity of the anterior spinal column, including biomechanical and biological functions. Endplate and IVD injuries are usually found in association with vertebral fractures (VFs); however, little is known about their relevance to the healing of osteoporotic VFs (OVFs). The first purpose of this study was to evaluate the incidence and occurrence pattern of endplate and IVD injuries associated with single- and acute-OVFs, and the second was to evaluate the influence of endplate and IVD injuries on the occurrence of delayed union.

**Methods:**

Endplate and IVD injuries associated with single- and acute-OVFs were retrospectively evaluated using magnetic resonance imaging (MRI). Vertebrae of 168 patients were included in the study. The occurrence rate and type of endplate and IVD injuries were radiologically evaluated, and the association between endplate and IVD injuries was statistically analyzed. Vertebrae of 85 patients, who received conservative treatment for acute OVFs, were included in the study and classified into two groups, union and delayed union, at 6 months after injury. To identify factors predicting delayed union, uni- and multivariate statistical analyses were performed. Vertebral MRI signal alternation patterns and endplate and IVD injuries were included as candidate factors in the logistic model.

**Results:**

In association with OVFs, endplate injuries were observed in 103 of the 168 vertebrae (61%), and IVDs lesions were observed in 101 of 168 OVFs (60%); the occurrence of both injuries was significantly associated. Although no significant association with endplate and IVD injuries was identified, multivariate analysis demonstrated that intravertebral signal alternation (focal high signal intensity) and posterior wall injury were independent risk factors that predicted delayed union.

**Conclusions:**

The results of this study showed that endplate and IVD injuries were found in approximately 60% of single and acute OVFs. These results suggest that fracture healing of OVFs would be mainly attributed to vertebral factors, including mechanical stress and metabolic status, among the three components of the anterior spinal column.

## Background

Vertebral fractures (VFs) are the most common skeletal injury resulting from osteoporosis within the increasing population of elderly individuals. Conservative treatments have generally been selected for osteoporotic VFs (OVFs), however the rate of non-union has been reported to range from 10 to 30% (see review in [1, 2]), and some patients require invasive surgeries because of progressive vertebral collapse and/or delayed neurologic deficit with deterioration of quality of life [3–5].

The healing of OVFs is considered to be influenced by the fracture site (vertebra), global alignment, bone strength (bone mineral density and bone quality), blood supply to the fractured vertebra, bone metabolism and other factors [6, 7]. Magnetic resonance imaging (MRI) signal alteration patterns within vertebral bodies (VBs) during the early phase of OVFs have recently been shown to predict an increased risk of delayed union [8–10].

Endplate and/or IVD injuries are usually found in association with VFs, however little is known about their relevance to the fracture healing of OVFs. The endplate-intervertebral disc (IVD) complex that is interposed between VBs is the basic unit of the anterior spinal column comprising spinal integrity [6, 11]. The endplates play a crucial role in maintaining the stress-strain relationship between adjacent vertebrae and support the integrity of IVD tissues [12–14]. The endplates, especially the cartilaginous layer, serve as a semipermeable interface that regulates nutritional transport from VB to IVD tissues [15]. Therefore, the endplate-IVD complex is closely interrelated with the VB in the structural integrity of the anterior spinal column, including biomechanical and biological functions.

Previous biomechanical studies have shown that damage to the VB and/or endplate altered the stress profiles within IVDs with high-stress concentrations in the posterior annulus [13, 14]. Conversely, the stress distribution of vertebrae has been shown to be dependent on the biomechanical properties of IVDs [16]. Therefore, the altered biomechanical properties of the IVD after an OVF with endplate injury are speculated to enhance the structural inconsistency of the spinal column. Therefore, we hypothesized that the occurrence of endplate and/or IVD injuries has a significant effect on the process of bone union following OVFs.

The first purpose of this study was to evaluate the incidence and occurrence pattern of endplate and IVD injuries evaluated by MRI after single and acute OVFs, and the second was to evaluate the effect of endplate and IVD injuries on the occurrence of delayed union after conservative treatment of OVFs.

## Methods

### Subjects

From January 2014 to March 2015, patients who were older than 60 years-old and diagnosed with an acute OVF of a single level of thoracic or lumbar vertebrae were reviewed at two different institutions (cohort A and B). An acute OVF was diagnosed on the basis of back pain or low back pain in a sitting position with MRI findings of low-intensity change on T1 weighted image (WI) and high-intensity change on MR T2 short-TI inversion recovery (STIR) images [17]. OVFs were differentiated from vertebral fractures caused by other pathological conditions such as spondylodiscitis and malignant tumors by their characteristic MRI findings [18, 19], blood test and physical findings.

In institution A (cohort A), MRI imaging on low-field open magnets at 0.3 T (Airis Elite, Hitachi, Tokyo) was used for evaluation of OVFs. The following sequences were obtained with 0.3-T MRI scanners: T1 weighted turbo spin echo with a slice thickness of 5 mm (repetition time [TR] 462 ms and time to echo [TE] 25.0 ms), the same sequence with T2 (TR 3203 ms and TE 110.0 ms), and STIR (a sequence with intrinsic fat saturation, TR 3320 ms, TE 80.0 ms, and inversion time 110 ms). One hundred six patients were reviewed for single and acute OVFs. Among them, 23 patients were excluded because of two continuous level fractures, VFs associated with diffuse idiopathic skeletal hyperostosis (DISH) and difficulty in radiological evaluation.

In institution B (cohort B), MRI imaging (Signa HDe, GE Healthcare Japan, Tokyo) was used for evaluation of OVFs. The following sequences were obtained with 1.5-T MRI scanners: T1 weighted turbo spin echo with a slice thickness of 5 (4/1) mm (repetition time [TR] 350–450 ms and time to echo [TE] 10–11 ms), the same sequence with T2 (TR 2300–3500 ms and TE 80— 100 ms), and STIR (a sequence with intrinsic fat saturation, TR 2000–4000 ms, TE 45–80 ms, and inversion time 120–170 ms). One hundred fifty-seven patients who received conservative treatment for acute OVFs were reviewed. Of these, 72 patients were excluded because of loss to 6 months clinical follow-up in addition to the exclusion criteria written above.

### Study design

#### Study 1

**Fig. 1:**
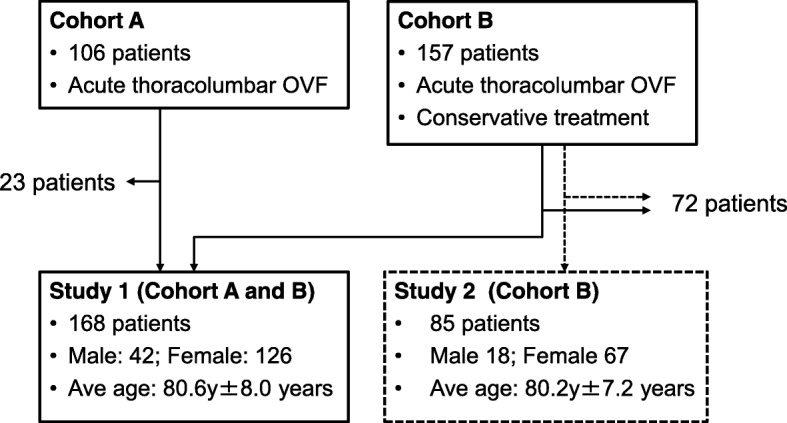
Study design. Acute osteoporotic vertebral fractures (OVFs) were evaluated using magnetic resonance imaging (MRI) of cohort A and B in Study 1. Prognostic factor analyses for delayed union were performed using MRI of cohort B in Study 2

Endplate and IVD injuries associated with single OVFs were radiographically evaluated using MRI of cohort A and B (Fig. 1). A total of 168 patients (168 VFs; 42 males, 126 females, average age 80.6 ± 8.0) were included in Study 1 (Fig. 1).

#### Study 2

Analyses of prognostic factors for delayed union after single OVFs were conducted with the subjects in cohort B. Eighty-five patients (85 vertebrae; 18 males, 67 females, average age 80.2 ± 7.2) were included in Study 2 (Fig. 1).

### Evaluation of endplate injuries associated with OVFs

**Fig. 2:**
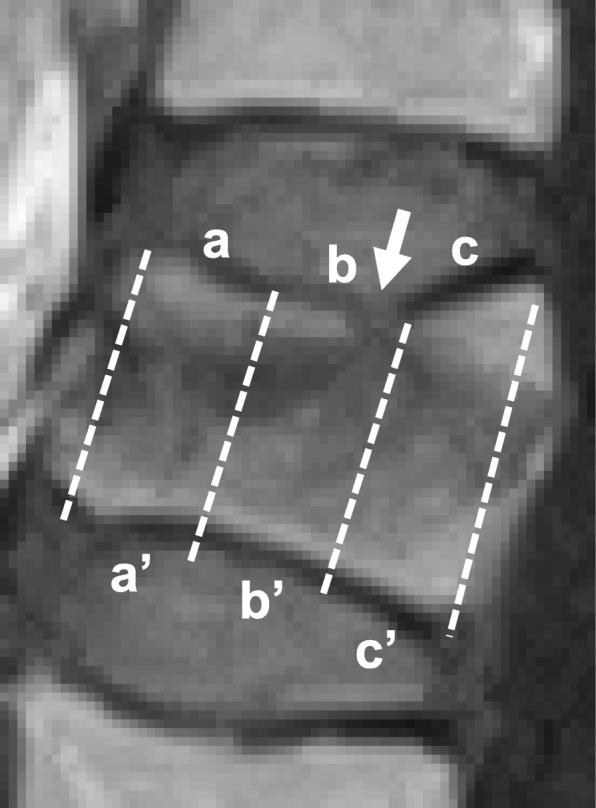
Distribution of endplate injuries. The location of endplate (EP) injuries was divided into three areas in sagittal MR T1-weight images (a and a’: anterior third of endplate, b and b’: middle third, c and c’: posterior third). Arrow indicates the location of EP injury

Endplate injuries were determined by the presence of cortical discontinuity or decisive angulation in sagittal MR T1-weight images based on Ortiz’s study with some modifications [20], and divided into three area of injury (anterior, middle and posterior third of endplate) (Fig. 2). Superior and inferior endplates adjacent to fractured vertebrae were evaluated respectively.

### Evaluation of IVD injury associated with OVFs

**Fig. 3:**
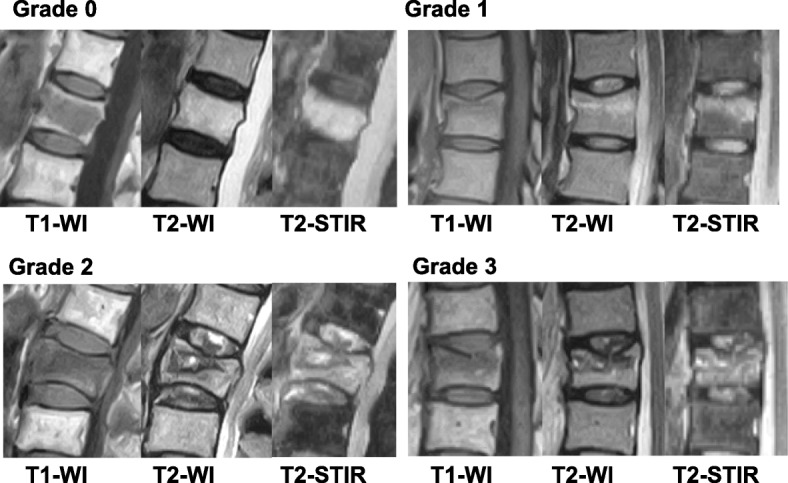
Signal alternation pattern of intervertebral disc (IVD) lesions. Sagittal MR T2 short-TI inversion recovery (STIR) images were divided into four types based on the signal alternation pattern (grade 0 to 3) as previously reported with modifications [21]. Grade 0: intact (no injury), grade 1: diffuse hyper-intense in T2 STIR images; grade 2: peri focal hyper-intense in T2 STIR images; grade 3: infraction of disc tissue into the endplate or the fractured vertebral body

IVD lesions adjacent to OVFs were evaluated by changes in signal intensity within a disc compared with normal adjacent levels in MR T2 STIR images [17]. IVD lesions were further divided into four types based on the signal alternation pattern (grade 0 to 3) as previously reported with some modifications [21] (Fig. 3). Grade 0 IVDs indicated no differences compared with uninjured discs adjacent to discs adjoining the OVF. Grade 1 IVDs included those with diffuse hyperintensity in T2 STIR images, which indicated tissue edema. Grade 2 IVDs were defined as those with perifocal hyper-intensity in T2 STIR images. Grade 3 IVD images included the infraction of the disc tissue into the endplate or the fractured VB.

### Intra- and inter-observer agreement assessment of endplate and IVD injuries

One orthopedic surgeon evaluated MRI images two times to assess intra-observer agreements. To assess inter-observer agreements, two orthopedic surgeons evaluated 30 randomly selected patients (30 vertebrae). Intra- and interobserver agreements were assessed by calculating the Cohen kappa coefficient. A kappa of < 0.00 was interpreted as minimal agreement, 0.00–0.20 as slight agreement, 0.21–0.40 as fair agreement, 0.41–0.60 as moderate agreement, and 0.61–0.80 as substantial agreement. The agreement was regarded as “substantial” when the kappa values were more than 0.61 [22].

### Prognostic factor analyses for delayed union following OVFs (study 2)

The diagnosis of delayed union was made on the basis of lateral radiographs of thoracic and lumbar spines with vacuum cleft and/or apparent vertebral collapse in a sitting position 6 months after injury, as previously reported [10]. The remaining patients without such radiographic findings were categorized as the union group. All patients in Study 2 were hospitalized in the acute phase as soon as possible after diagnosis of OVF. The patients were placed on bed rest for 2 weeks and then started exercising to return home and/or to society with a soft brace [23]. Patient characteristics, including age, gender, spinal level of OVFs, and MRI findings at the acute phase, were reviewed (Table 4).

The prognostic factor patterns of intensity changes within fractured VBs on 85 MR images were classified on both mid-sagittal T1- and T2-weighted images, as previously reported [10]. The intensity changes on T1-weighted images were classified into two patterns: diffuse low-intensity type and confined low-intensity type [10]. T2-weighted images were also classified into five patterns: confined high-intensity type, diffuse high-intensity type, confined low-intensity type, diffuse low-intensity type and normal type, as previously reported [10]. Middle column injury was evaluated by protrusion of the posterior wall of the VB into the spinal canal, as previously reported [24].

### Statistical analysis

In Study 1, the association between endplate injuries and those of their location, IVD injury types and their location, and the simultaneous occurrence of endplate and IVD injuries were statistically analyzed by the Chi-square test or Fisher’s exact test for categorical variables.

In Study 2, univariate analyses (Chi-square test or unpaired t-tests) were performed to examine the association between baseline characteristics and delayed union of OVFs. The association between delayed union and age, gender, spinal level, T1-WI classification, T2-WI classification, posterior wall (PW) injury, and endplate injury or IVD injury was statistically assessed by the Chi-square test or Fisher’s exact test followed by a post hoc test. The post hoc test was performed to assess the probability values for each combination of independent category levels by using a Bonferroni correction to control for type I error inflation [25–27]. To elucidate prognostic factors for delayed union at 6 months, a multivariate statistical analysis was performed. Factors included in the multivariate model were age, sex, spinal level of fracture, MRI images of vertebral body signal alteration pattern at T1WI (diffused low) and T2WI (diffused low, focal high), posterior wall (PW) injury, endplate injury, and IVD lesion. Odds ratios (ORs) and 95% confidence intervals (CIs) for the occurrence of delayed union were calculated as approximations of the relative risk estimates. Significant differences were evaluated with a *P*-value of <0.05. All analyses were performed using SPSS (IBM Japan, Tokyo).

## Results

### Intra- and inter-observer agreement assessment

Intra- and inter-observer agreements for the assessment of endplate injuries were “substantial” with kappa values of 0.88 and 0.70 and % agreements of 86.7 and 85.2%, respectively. In the assessment of IVD injuries, intra- and inter-observer agreements were also “substantial” with kappa values of 0.74 and 0.62 and % agreements of 81.5 and 80.7%, respectively.

### Distribution of OVFs

**Fig. 4:**
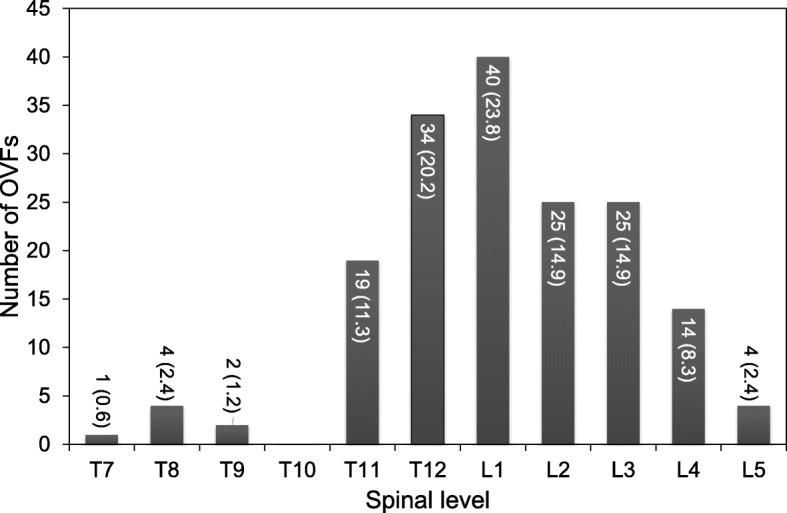
Distribution of osteoporotic vertebral fractures (OVFs). The number of OVFs in each spinal level in Study 1 is indicated with the percentage of the total number (168) of OVFs indicated in parentheses

A total of 168 single level OVFs were evaluated in this study. Among these, the number of OVFs was highest at the L1 level (40 OVFs, 23.8% of the total) followed by T12 (34 OVFs. 20.3% of the total), L2 and L3 (25 vertebrae, 14.9% of the total, respectively) and T11 (19 OVFs, 11.3% of the total) (Fig. 4). Seven OVFs (4.2% of the total) were found in thoracic levels, 118 OVFs (70.2% of the total) in thoracolumbar levels and 43 OVFs (25.6% of the total) in lumbar levels.

### Endplate injuries associated with OVFs

**Fig. 5:**
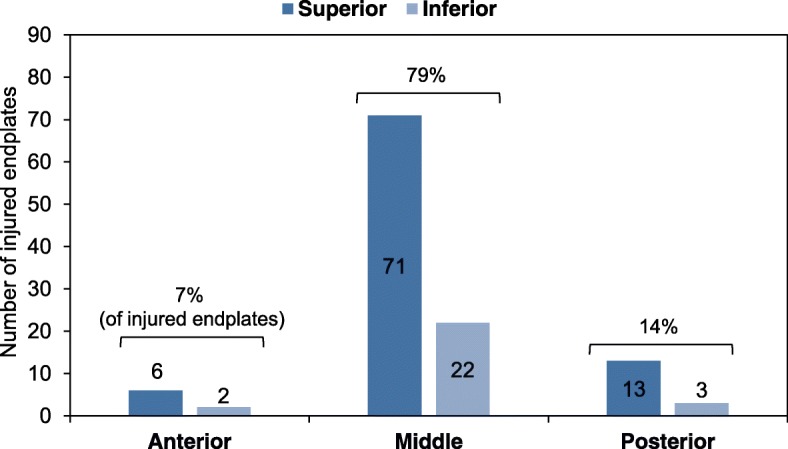
Incidence of endplate injury in osteoporotic vertebral fractures (OVFs). The numbers of injured endplates in the anterior, middle and posterior third of the endplate in both superior and inferior endplates are indicated in the graph. The percentage of endplate injuries (sum of superior and inferior endplates injuries) of the three areas are indicated above the bracket

In association with OVFs, endplate injuries were observed in 103 of the 168 vertebrae (61% of total number of OVFs) (Table 1). The incidence of endplate injuries was higher in superior endplates (39% of total) than in inferior endplates (5%). Simultaneous endplate injuries in both the superior and inferior endplates were also found in 17% of total OVFs (Table 1). When endplate injuries were analyzed into three sagittal distributions, the middle third of the endplate was more common (93 endplates, 79%) followed by the posterior third (16 endplates, 14%) and the anterior third (8 endplates, 7%) (Fig. 5). There were no significant differences in the distribution of endplate injuries between superior and inferior endplates (*P* = 0.904).

**Table 1:** Incidence of endplate injury in osteoporotic vertebral fractures (OVFs)

Endplate injury	Number	Percentage (%)
Intact	65	39
Superior only	66	39
Inferior only	8	5
Both	29	17
Total	168	

The number and percentage (%) of the total number of OVFs is indicated

### Intervertebral disc injuries associated with OVFs

High signal intensity changes of MRI (MR T2 STIR images) in the superior and/or inferior IVDs adjacent to OVFs were observed in 101 of 168 OVFs (60%) (Table 2). Among these, signal changes in both the superior and inferior IVDs adjacent to OVFs were the most common (27% of the total OVFs), followed by superior only (23%) and inferior only (10%) (Table 2).

**Table 2:** Incidence of intervertebral disc (IVD) injury in osteoporotic vertebral fractures (OVFs)

IVD	Number	Percentage
Intact	67	40
Superior only	39	23
Inferior only	16	10
Both	46	27
Total	168	

Percentage (%) to the total number of OVFs is indicated

**Fig. 6:**
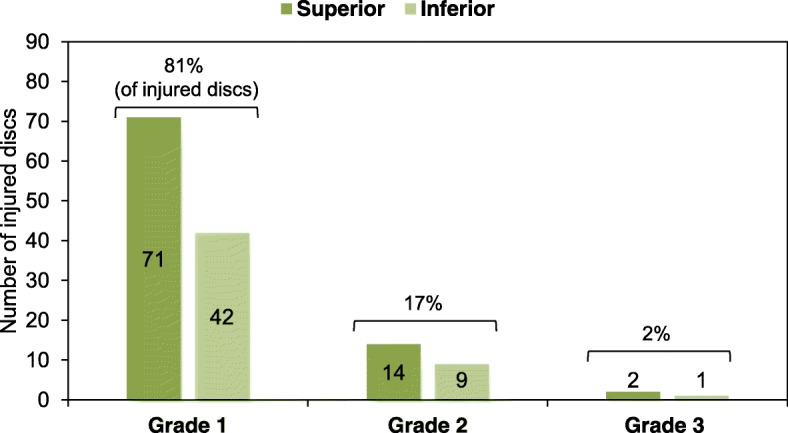
Incidence of intervertebral disc (IVD) lesion in osteoporotic vertebral fractures (OVFs). The numbers of injured discs adjacent to OVFs in both the superior and inferior sides in each grade of IVD injuries are indicated in the graph. The percentage of IVD injuries (sum of superior and inferior IVD injuries) of each grade is indicated above the bracket

Furthermore, focusing on the patterns of signal changes within the IVD, grade 1 IVD lesions were more common (113 discs, 81% of injured discs) followed by grade 2 (23 discs, 17%) and grade 3 (3 discs, 2%) (Fig. 6). No significant differences were found in the occurrence of these three signal change patterns between superior and inferior IVDs (*P* = 0.974).

### Association between endplate injuries and intervertebral disc injuries

The Chi-square test showed that the incidence of IVD injuries was significantly associated with endplate injuries (*P* < 0.001) (Table 3). IVD injuries were more frequently observed in cases with endplate injuries (*n* = 74) than in cases with no endplate injuries (*n* = 27). Next, the effect of endplate injury on the MRI-signal pattern of IVD lesions was evaluated in OVFs with IVD lesions. Statistical analysis showed that endplate injury had no significant effect on the MRI-signal pattern of IVD lesions (*P* = 0.80).

**Table 3:** Association between endplate and intervertebral disc (IVD) injuries

	Disc intact	Disc injury
Endplate intact	38 (23%)	27 (16%)
Endplate injury	29 (17%)	74 (44%)

Percentages of the total number (168) of osteoporotic vertebral fractures (OVFs) are indicated in parentheses. The numbers of the incidence of IVD injuries (disc injury) are indicated with or without endplate injury, respectively

### Risk factor analysis for delayed union at six months after treatment of OVFs

After conservative treatment in institution B (cohort B) for 85 acute OVFs, 68 (80%) patients had bone union while 17 (20%) patients were diagnosed as having “delayed union.” Univariate analysis demonstrated that spinal level (focal high signal intensity on T2WI) and posterior wall injury were significantly associated with delayed union at 6 months post-injury (Table 4).

**Table 4:** Characteristics of participants in Study 2

	Union	Delayed union	*P*-value
Number of OVFs	68 (80)	17 (20)	
Age	80.5 (±7.3)	78.8 (± 6.9)	0.394
Gender (male/female)	15/53	3/14	0.690
Spinal level			0.133
Thoracic spine	2 (66.7)	1 (33.3)	
Thoracolumbar spine	41 (74.5)	14 (25.5)	
Lumbar spine	25 (92.6)	2 (7.4)	
T1-WI			0.693
Diffuse low	62 (79.5)	16 (20.5)	
Focal low	6 (85.7)	1 (14.3)	
T2-WI			**<0.001**
Focal high	7 (43.8)	9 (56.3)	**<0.001**
Focal low	24 (88.9)	3 (11.1)	
Diffuse high	14 (100)	0 (0)	
Diffuse low	23 (82.1)	5 (17.9)	0.729
PW injury			**0.003**
Intact	43 (63.2)	25 (36.8)	
Injured	4 (75.6)	13 (24.4)	
Endplate injury			0.329
Intact	37 (84.1)	7 (15.9)	
Injured	31 (75.6)	10 (24.4)	
IVD injury			0.121
Intact	30 (82.2)	4 (11.8)	
Injured	38 (74.5)	13 (25.5)	

Within each group, age is expressed as mean with standard deviation (SD) indicated in parenthesis. T1-weighted image classification (T1-WI), T2-weighted image classification (T2-WI), posterior wall injury (PW injury), endplate injury and intervertebral disc (IVD) injury, the number in parentheses indicates the % of the total number (union plus delayed union) of osteoporotic vertebral fractures (OVFs) that fall within each category. Bolded numbers indicate statistically significant *P*-value numbers

The multivariate analysis demonstrated that focal high signal intensity on T2WI and posterior wall (PW) injury were independent risk factors for the occurrence of delayed union at 6 months post-injury (Table 5).

**Table 5:** Risk factors for delayed union (multivariate analyses)

	Odds Ratio	95% CI	*P* value
T2-WI (focal high)	9.0	2.4–33.4	0.001
PW injury	5.1	1.3–19.1	0.016

T2-WI: T2-weighted image (T2WI), PW: posterior wall

### Association between endplate injury and MRI findings of vertebral lesions that predict delayed union

**Fig. 7:**
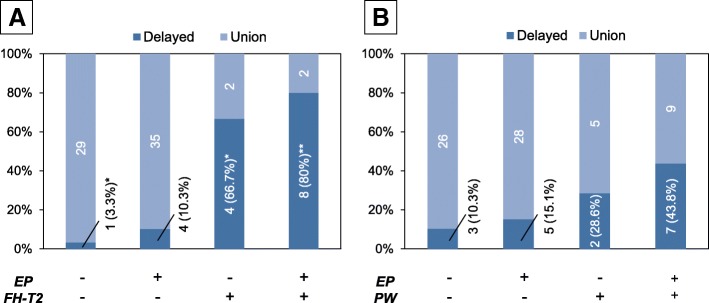
Effect of coincidence of endplate injury with vertebral lesions that predict delayed union of fracture healing. The percentage of delayed union (delayed) with or without endplate (EP) injury in the presence or absence of FH-T2 in osteoporotic vertebral fractures (OVFs) (**a**), and the percentage of delayed union with or without endplate injury in the presence or absence of posterior wall (PW) injury in OVFs (**b**). The numbers of OVFs in each category are indicated in the columns of the graph. The percentage of delayed union in each group is indicated in parentheses

Statistical analysis showed that there was no significant association between MRI findings of endplate injury and focal high intensity on T2WI (FH-T2) (*P* = 0.170). Next, to evaluate whether endplate injury has additional effects on delayed union of single-level OVFs, the percentage of delayed union in the presence or absence of risk factors for FH-T2 with or without endplate injury was evaluated (Fig. 7a). In OVFs without both endplate injury and FH-T2, the percentage of patients with delayed union was only 3.3%; that increased to 10.3% in those cases with endplate injury only. That percentage increased to 66.7% with MRI finding of FH-T2 only and further increased to 80% with MRI findings of both FH-T2 and endplate injury (80.0%). Fisher’s extract test showed that the occurrence of delayed union was significantly associated with MRI findings with endplate injury and/or FH-T2 (*P* < 0.001). The post-hoc test revealed that the occurrence of delayed union was significantly lower in OVFs without MRI findings of both FH-T2 and endplate injury (*P* = 0.006). The occurrence of delayed union was significantly higher in OVFs with FH-T2 findings either with or without endplate injuries (P = 0.006 and P < 0.001, respectively).

The Chi-square test showed that there was a significant correlation between the occurrence of endplate injuries and posterior wall (PW) injuries (*P* = 0.020). When the percentage of delayed union was evaluated with or without endplate injury in the presence or absence of PW injury, the percentage was highest (43.8%) in the OVFs with endplate and PW injuries group, followed by the PW injury only group (28.6%), the endplate injury only group (15.1%) and those without both findings (10.3%) (Fig. 7b). The Fisher exact test showed that there was a significant correlation between the occurrences of delayed union and MRI findings of endplate and/or PW injuries (P = 0.020), although the post-hoc test showed no statistical significance on each column.

### Association between IVD lesions and MRI findings of vertebral lesions that predict delayed union

**Fig. 8:**
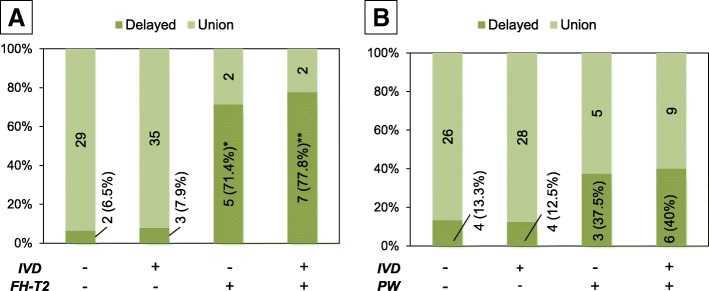
Effect of coincidence of intervertebral disc (IVD) injuries with vertebral lesions that predict delayed union of fracture healing. The percentage of delayed union with or without IVD injuries in the presence or absence of FH-T2 in osteoporotic vertebral fractures (OVFs) (**a**), and the percentage of delayed union with or without IVD injuries in the presence or absence of posterior wall (PW) injury in OVFs (**b**). The number of OVFs in each category is indicated in the columns of the graph. The percentage of delayed union in each group is indicated in parentheses

The Chi-square test showed there was no significant correlation between MRI findings of IVD injury and FH-T2 (*P* = 0.875). The percentage of delayed union was highest (77.8%) in OVFs with MRI findings of both IVD lesions and FH-T2, followed by FH-T2 only (71.4%), IVD lesions only (7.9%) and those without both findings (6.5%) (Fig. 8a). Fisher’s exact test showed that there was a significant association between the occurrence of delayed union and MRI findings of IVD lesions and/or FH-T2 (*P* < 0.001). The post-hoc analysis revealed that a significantly higher occurrence rate was found in MRI findings of OVFs of the FH-T2 only group (*P* = 0.006) and of OVFs with both IVD lesions and FH-T2 group (*P* < 0.001).

Lastly, statistical analysis showed no significant association between IVD lesions and PW injury. The percentage of delayed union was highest in OVFs with MRI findings of IVD lesions and PW injury (40%), followed by PW injury only (37.5%), IVD injury only (12.5%) and those without both findings (13.3%) (Fig. 8a). However, Fisher’s exact test revealed no significant association between the occurrence of delayed union and MRI findings of IVD lesions and/or PW injury.

## Discussion

Considering the pathophysiology of OVFs as an injury of the anterior spinal column, which consists of the vertebral body and its surrounding structures, we paid special attention to the endplate and IVD injuries associated with OVFs. The current study retrospectively evaluated MRIs of acute OVFs and showed that vertebral endplate and adjacent IVD injuries were frequently associated with OVFs. Endplate injuries were found in 61% of OVFs and commonly occurred on the superior side and middle third of the endplate. IVD injuries were identified in 60% of OVFs and frequently occurred on the superior side with a diffused signal alteration pattern (grade 1). Multivariate analysis showed that intravertebral signal alternation (focal high signal intensity) and posterior wall injury were independent risk factors that predict delayed union, although no significant association with endplate and IVD injuries was identified.

Ortiz et al. [20] reported a high incidence of endplate injuries (80%) associated with OVFs. The frequency (endplate injury 61%) was lower in our study than in the Ortiz report. In our study, endplate injuries were defined as remarkable morphological changes on MRI T1 images focusing on cortical discontinuity or decisive angulation; however, endplate edema or fluid collection [20] was not included. These differences in the definition of endplate injuries would be responsible for the discrepancy in the results.

The results of our study and that of Ortiz showed that superior endplate injuries were more common than inferior endplate injuries. Furthermore, our study revealed that the injuries to the middle third of the endplate were more common than any other distribution for both superior and inferior endplates. A previous biomechanical study using cadaveric motion segments showed that endplate strength and stiffness were highest in the postero-lateral and lowest in the center of the endplate [28]. Another mechanical study using micro-CT-based finite element analysis showed that the initial failure of an osteoporotic vertebra was associated with high tensile stress in the endplate and that the typical distribution of high strain in endplates was more concentrated in the central region of superior endplates than in inferior endplates [29]. These previous studies would explain the occurrence pattern and distribution of endplate injuries associated with OVFs.

The frequency of IVD injuries associated with OVFs was lower in our study (60%) than in the Ortiz report (79%) [20]. Ortiz et al. evaluated the IVD injuries by the presence of edema or morphological alternation on MRI. On the other hand, in our study, IVD lesions were evaluated and classified by MRI (T2-STIR) according to Sander’s report with some modifications [21].

Ghanem et al. [30] evaluated MRIs and discography of injured IVDs adjacent to VFs in trauma patients and found positive concordant imaging findings of IVD injuries by both MRI and discography in 54% (29/54) of cases. Among these, a signal increase in T1-WI was found in the majority of cases (69%), suggesting that MRI findings of IVD injury include intradiscal bleeding. On the other hand, our results showed that a signal increase in T1-WI was identified in 8.7% (2/23) cases among grade 2 disc lesions (data not shown). Therefore, grade 2 discs in our OVF study would indicate tissue fluid or edema eccentrically located within injured IVDs, but less intradiscal bleeding.

In Sander’s report [21] of 102 patients (average age: 42.9 years) with trauma-induced single-level thoracolumbar fractures, IVD lesions were found in 71.1% of cases, with grade 3 being the highest (40.7%), followed by grade 2 (25.5%) and grade 1 (4.9%); the order of occurrence rate in each grade was opposite to that of our OVF study. Compared to traumatic VFs, OVFs are considered to occur with low-grade energy injuries; this might be reflected in the opposite occurrence rate in the grading of IVD lesions between traumatic VFs and OVFs (Fig. 7).

In our study, among the injured discs following an OVF, 73.3% (74/101) of the IVDs were accompanied by endplate injuries, suggesting that the vast majority of IVD injuries occurred in response to the endplate injury. Previous in vitro studies have shown that an endplate injury reduced pressure in the nucleus and altered the mechanical properties of adjacent IVDs [13, 14]. These previous studies led us to speculate that an endplate injury would not only break down the barrier function between vertebrae and IVDs, but also change endplate diffusibility, leading to changes in the transfer of water and solutes into adjacent IVDs that were expressed by signal alterations of IVDs following OVFs.

In previous studies, the risk factors for delayed union or vertebral collapse have been reported to be posterior wall injury [10, 31, 32], steroid use [31], hyper-intense limited variations and hypo-intense wide variations in T2WI of MRI [9, 31], thoracolumbar fracture [10, 32], decreased bone mineral density [32], prior bisphosphonate use [32] and advanced age [23]. Although we could not make easy comparisons with these studies because of different conservative treatment strategies, outcome settings and patient characteristics, the risk factors that predict delayed union of OVFs in the current study were consistent with those of previous studies [9, 10].

Rahmani et al. [33] have recently evaluated whether endplate fracture (injury) and adjacent disc degeneration have a significant association with the occurrence of delayed union following OVFs. They found that an earlier stage of disc degeneration at the caudal level and endplate injuries (both cranial and caudal sides) were significantly associated with an increased risk of delayed union. In their study, endplate fracture (injury) was determined based on the irregular image of the endplate and signal change in the adjoining bone marrow of vertebrae in sagittal MR T1-weight images. They identified endplate injury in almost all OVF patients and the occurrence rate of endplate injury was considerably higher than ours and that of a previous study [20], probably because of differences in the definition of endplate injury. Rahmani and colleagues also evaluated the signal changes of adjacent IVDs in MR T2-weighted images at enrollment and 6 months follow-up based on a modified Pfirrman grading system; however, the signal changes resulting from IVD injuries (associated with endplate injuries) had not been evaluated [33]. Differences in the definition of endplate injury and IVD lesions may be responsible for the discrepancy between the results of risk factor analyses of delayed union between their study and ours.

Our statistical analyses revealed that the occurrence of endplate or IVD injuries has little influence on the occurrence of intravertebral FH-T2 signal; this suggests that the occurrence of intravertebral FH-T2 signal would be affected by repeated mechanical stress directly to the intravertebral fractured site [10], but not by surrounding components.

We next evaluated the effect of endplate injury or IVD injury, in addition to intravertebral FH-T2 signal, on the occurrence of delayed union. Interestingly, the additional MRI finding of endplate injury or IVD injury to intravertebral FH-T2 had a tendency to increase the occurrence rate of delayed union, compared to that of intravertebral FH-T2 only.

Endplate injury had a significant influence on the occurrence of PW injury, while IVD injury had no significant influence on its occurrence. Tawara et al. [34] reported, in a biomechanical study, that fragile vertebra with more structural damage had repercussions on the posterior wall, which was consistent with our results that endplate injury was associated with PW injury in cases of OVFs.

There are several limitations to this study. First, this study was a retrospective observational study in which patients had variations in bone mineral density, past OVF history, pharmaceutical agent use, duration between onset and hospital admission, and timing of MRI examination with a potential selection bias. Another limitation was small sample size. Most OVFs were successfully classified into the union group; therefore, only 17 vertebrae fell into the delayed union group in Study 2. Further investigation with a larger sample size is needed to validate the results of the study reported here.

## Conclusions

In this study, we have embraced the concept that OVFs are not only intravertebral lesions but also lesions of the vertebra, endplate and IVD complex, the basic unit for the spinal integrity of the anterior spinal column. The present results suggest that the occurrence of delayed union could be mainly attributed to vertebral factors among the three components of the anterior spinal column. Further studies are needed to evaluate the clinical implication of endplate and IVD injuries, including the generation of pain or vertebral deformity accompanying OVFs.

## Data Availability

The dataset supporting the conclusions of this manuscript is included within the manuscript.

## Abbreviations

AF: annulus fibrosus
CT: computed tomography
DHI: disc height index
IVD: intervertebral disc
LSS: lumbar spinal stenosis
MDCT: multi-detector computed tomography
MRI: magnetic resonance imaging
NP: nucleus pulposus
VP: vacuum phenomenon
